# Descriptive retrospective study analyzing relevant factors related to dental implant failure

**DOI:** 10.4317/medoral.23082

**Published:** 2019-10-27

**Authors:** Lizet Castellanos-Cosano, Antonio Rodriguez-Perez, Sergio Spinato, Marcel Wainwright, Guillermo Machuca-Portillo, Maria Angeles Serrera-Figallo, Daniel Torres-Lagares

**Affiliations:** 1 Associate Professor. Oral Surgery, School of Dentistry, University of Seville. University of Fernando Pessoa Canarias; 2 Full-time Professor, School of Dentistry, University of Fernando Pessoa Canarias; 3 Private Practice in Dentistry; 4 Full-time Professor, School of Dentistry, University of Seville

## Abstract

**Background:**

The objective of this retrospective descriptive study was to analyze the characteristics of incident reports provided by dentists while using a specific brand of dental implants.

**Material and Methods:**

The study was carried out in collaboration with Oxtein Iberia S.L.®, with the company providing access to the incident database in order to evaluate the characteristics of incidents from January 2014 to December 2017 (a total of 917 over four years). The data sheet recorded different variables during each of the stages of implant treatment, from initial implant placement to subsequent prosthetic rehabilitation. These variables included age, sex, systemic pathologies, smoking habits, bone quality, implant type, prosthesis type, and type of load applied, among others. SPSS Statistics was used to perform statistical analysis of the qualitative variables (univariate logistic regressions, χ2 test, Haberman's adjusted standardized residuals).

**Results:**

The total study sample consisted of 44,415 implants shipped from Oxtein® warehouses on the dates indicated, of which 917 implants (2.1%) were flagged due to reports of lack of primary stability, failed osseointegration, or implant failure within one year of placement. When analyzing incident reports, it was observed that 61.6% of incidents occurred in male patients, compared to 38.4% in female patients. The average age of patients in the reported cases was 56.12 ± 12.15 years. A statistically significant correlation was discovered between incidents of implant failure and tobacco use, diabetes, heart disease, poor oral hygiene, previous infection, poor bone quality, and bruxism (*p* < 0.05). A (statistically significant) higher rate of incidents was also observed in tapered, internal connection, Grade IV titanium, narrow, and short implants.

**Conclusions:**

Analysis of these implants reveals a higher rate of complication in short, tapered, internal connection and narrow-diameter implants. These data can help and encourage clinicians to use the utmost surgical precautions when placing these implants.

** Key words:**Pharmacovigilance, Dental implant, Dental implant failure.

## Introduction

Oral rehabilitation with dental implants has been described as a predicTablealternative treatment with a success rate higher than 90% (1) to other alternatives for prosthetic treatment (fixed dental prosthesis, removable prosthesis). Even though treatment with dental implants is predicTable, it is not without its complications, the most prevalent of which are peri-implant mucositis (19–65%), peri-implantitis (1–47%) (2), esthetic failures, and complete loss of osseointegration prior to functional loading (3).

Healthcare products from the Spanish Agency of Medicines and Medical Devices (AEMPS) are subject to regulations as per Regulation (4) 2017/745 of the European Parliament. Healthcare products include a monitoring system that reports adverse effects, whether caused either by the product itself, its registration and evaluation, the adoption of appropriate measures to protect health, or the communications of these measures to the relevant parties.

The recent Regulation (4) 2017/745 has increased product safety through a new post-marketing oversight scheme that requires manufacturers to document and maintain a monitoring system of products after placing them on the market, using a plan that reflects risk level and product type. This obliges manufacturers to periodically report the results and conclusions of their analyses throughout the lifetime of the product. This oversight scheme is of vital importance not just for the device manufacturers, but also for consumers and the dentists involved in the placement of dental implants.

Nevertheless, the scientific literature lacks any published studies with an exhaustive analysis of the variables recorded in these dental implant incident reports. The aim of the present study was to address the following question: Which variables are most frequently observed alongside the occurrence of complications and/or dental implant failure in the incident reports provided?

## Material and Methods

A retrospective descriptive study was carried out to analyze the variables linked to the occurrence of complications and/or dental implant failure. Oxtein Iberia S.L.® collaborated with the study, providing access to its company database of incidents, as well as the total number of implants shipped from their warehouses, in order to evaluate the characteristics of incident reports submitted between January 2014 and December 2017 (four years).

The data sheet included different variables throughout the various stages of the implant treatment process, ranging from initial implant placement up to subsequent prosthetic rehabilitation over the following year. The data was used anonymously, and consent was obtained for its use in this way. This study was authorized by the Research Ethics Committee of the Virgen del Rocío - Macarena University Hospitals (USE-2018-1401).

This study analyzed sociodemographic variables related to the patient, including age and sex, medical history (psychological conditions, bruxism, drug use (recognized addiction and excessive consumption of drugs, including alcohol), smoking habits (patient with a consumption of more than five cigarettes a day was considered a smoker), infectious diseases (HIV and other infectious conditions that may affect the patient's immune response), hygiene (poor hygiene was considered when the patient does not meet at least two daily brushes associated with bad plaque control), diabetes (controlled, in treatment), cardiovascular pathologies (including hypertension if it is being treated with drugs), blood dyscrasias, blood clotting disorders), dental history (presence of periodontal pathologies, previous antibiotic treatment), variables related to dental implants (implant position, implant type, type of connection, grade of titanium, implant diameter, implant length, type of loading), and other dental aspects (bone quality (Type I to IV, classic classification of Lekholm and Zarb), implant nonparallelism, postoperative infections, post-extraction implant placement, implant placement performed in conjunction with sinus lifting and including biomaterials).

**Figure 1:**
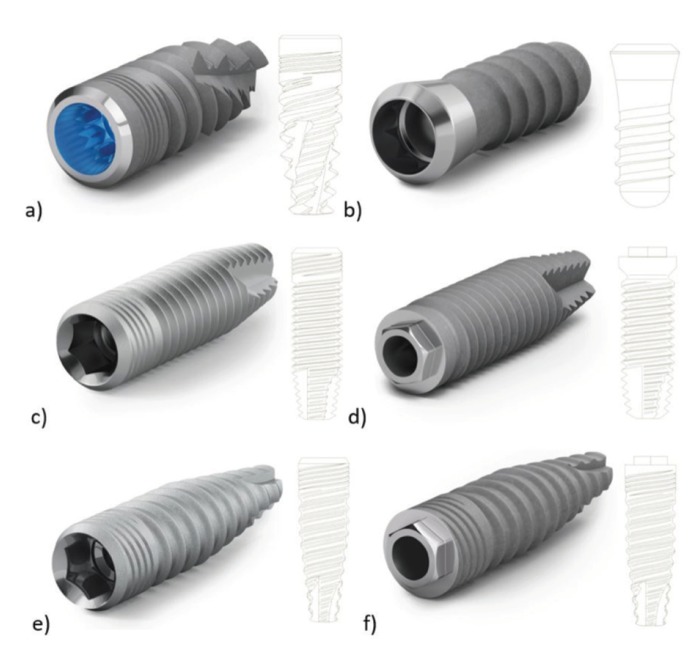
Images of the implants used in the present study. a) M12: Morse taper connection, aggressive tapered profile, Grade IV titanium; b) M8: Internal octagon connection, less aggressive cylindrical profile, Grade IV titanium; c) L35: Internal hexagon connection, cylindrical profile, Grade V titanium; d) L6: External hexagon connection, cylindrical profile, Grade V titanium; e) N35: Internal hexagon connection, tapered profile, Grade V titanium; f) N6: External hexagon connection, tapered profile, Grade V titanium.

Fig. 1 shows the characteristics, design, and connection type of the implants included in the study.

Implant position was classified into four categories: anterior maxilla, which includes the upper central incisor, upper lateral incisor and upper canine (UCI, ULI y UC); posterior maxilla, which includes the upper molars and premolars (UP and UPM); anterior mandible, which includes the lower central incisor, lower lateral incisor and lower canine (LCI, LLI and LC); and posterior mandible, which includes the lower molars and premolars (LM and LPM).

- Statistical Analysis.

SPSS Statistics was used to perform statistical analysis using univariate logistic regressions, χ2 tests, and crosses between the variables to determine the statistical significance of the differences for qualitative variables. Haberman's adjusted standardized residuals were used to determine which groups presented significant differences, enabling the significance of each criterion to be calculated independently.

For the quantitative variables, crosses were made and the normality test showed that not all of the analyzed variables follow a normal distribution (Kolmogorov–Smirnov test). Therefore, the results of the corresponding non-parametric tests were considered when determining statistical significance (Mann-Whitney U for the cross of dichotomous variables, or Kruskal Wallis to determine the general significance between variables with more than two categories). In addition, when a test was significant, the Mann-Whitney U was used for comparisons between groups (two by two), and the groups that made the difference between them were determined.

A correlation was deemed statistically significant for values of *p* < 0.05 (p < 0.01; p < 0.001, p < 0.0001, and p < 0.00001); the lower the number, the higher the statistical significance. Furthermore, the word "quasi" is indicated in gray when the result is almost but not quite statistically significant (0.05 ≤ p < 0.10).

## Results

The total study sample consisted of 44,415 implants placed during this period, of which 917 implants (2.1%) were flagged up in incident reports due to lack of primary stability, failed osseointegration, or implant failure within one year of placement.

The average age of patients in the given incident reports was 56.12 years ± 12.15, with a higher frequency of men (61.6%) than women (38.4%). Table 1 shows general details provided in the incident reports of implant failure as categorized according to the studied variables.

**Table 1:**
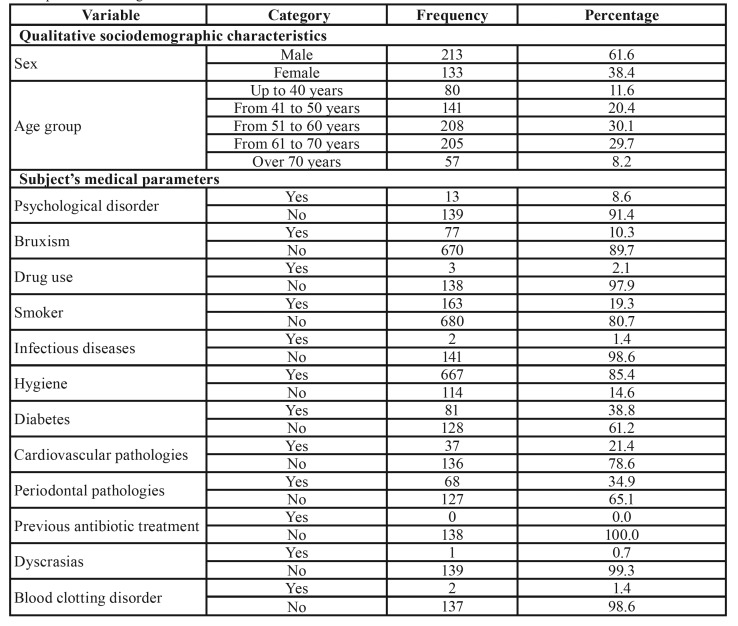
General characteristics of the sample. Note: If the sum of the categories does not reach the value of 917, the differential corresponds to missing data.

Table 2 and Table 2 cont. provides a detailed analysis of the recorded variables with regard to incident reports of implant failure categorized by sex. A higher frequency was found in male smokers (p < 0.01), men with poor oral hygiene (*p* < 0.05), men with internal connection implants that had failed (*p* < 0.05), men with more implants placed after tooth extraction (*p* < 0.05), as well as a higher failure rate involving two implants (*p* < 0.01). However, female patients were more likely to have had an implant placed immediately after sinus lifting (*p* <0.05), women with external connection implants (*p* < 0.05), and a higher percentage of incidents of failure of one implant (*p* < 0.01).

With regard to age, the incident reports were categorized according by age group: up to 40 years (11.6%), 41 to 50 years (20.4%), 51 to 60 years (30.1%), 61 to 70 years (29.7%), and over 70 years old (8.2%). No statistically significant differences between age groups were found between men and women or the different patient groups.

**Table 2:**
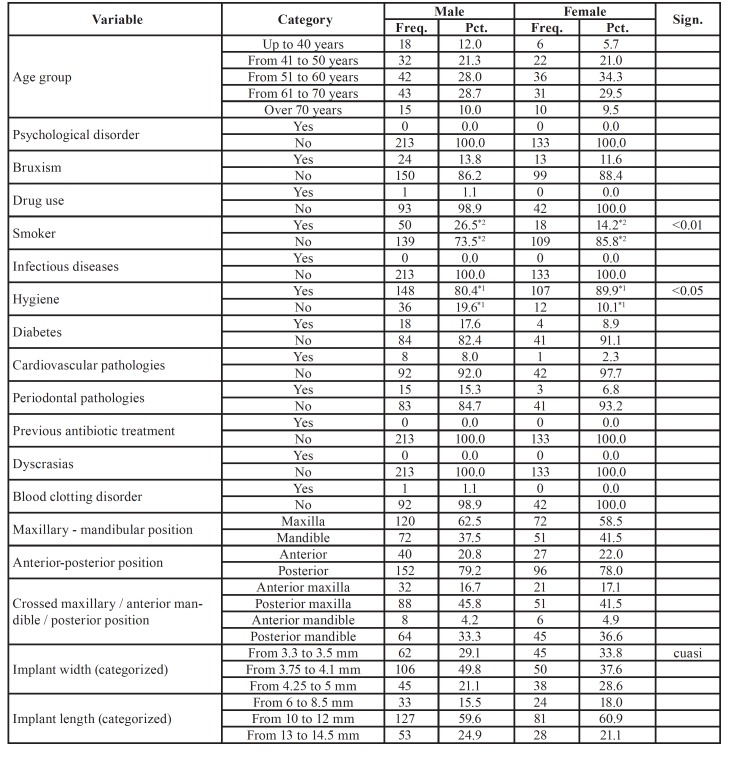
Characteristics according to sex. Note: If the sum of the categories does not reach the value of 917, the differential corresponds to missing data.

**Table 2 cont.**
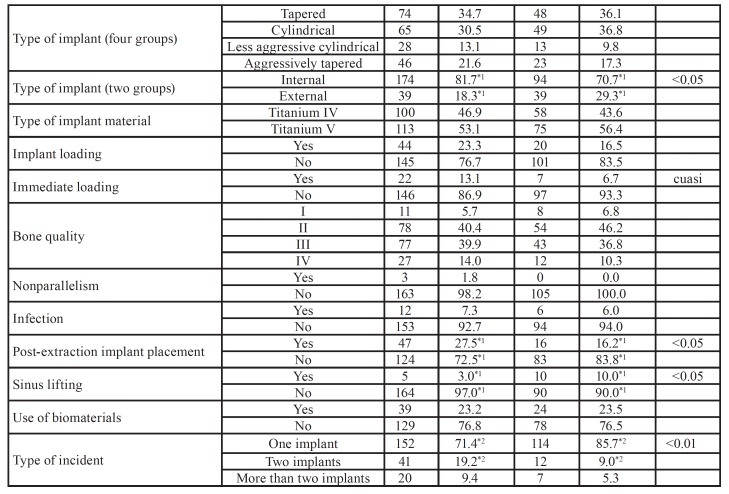
Characteristics according to sex. Note: If the sum of the categories does not reach the value of 917, the differential corresponds to missing data.

Table 3 and Table 3 cont. show the analysis of the studied variables in relation to incident reports of failed implants as categorized according to age groups. The reports in which the age of the patient was between 51–60 years old showed a greater frequency of patients with Type IV bone (p < 0.05), a higher percentage of postoperative infections (p < 0.05), the highest percentage of implants placed immediately after sinus lifting (p < 0.001), and the highest rate of incidents involving two or more implants (p < 0.01) in comparison with patients between 61 and 70 years of age (p < 0.05). Incident reports involving patients aged between 61 and 70 showed greater frequency of cardiovascular pathologies (p < 0.001), patients with worse oral hygiene (p < 0.05), a higher frequency of Type III bone (p < 0.05), and the highest amount of implants placed after tooth extraction (p < 0.01), as well as the highest percentage of implants place with immediate loading (p < 0.01). In the case of reports involving patients older than 70, these showed a higher percentage of diabetes mellitus (80%; p < 0.001) and blood clotting disorders (40%; p < 0.0001).

**Table 3:**
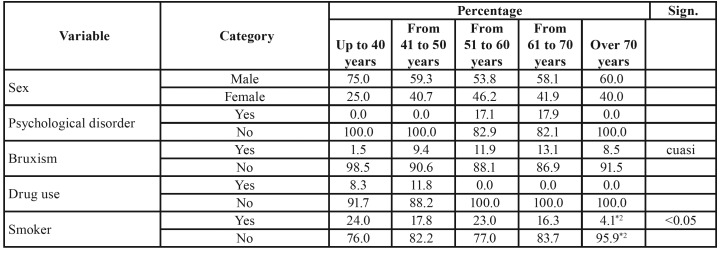
Crosstab analysis of age. Note: If the sum of the categories does not reach the value of 917, the differential corresponds to missing data.

**Table 3 cont.**
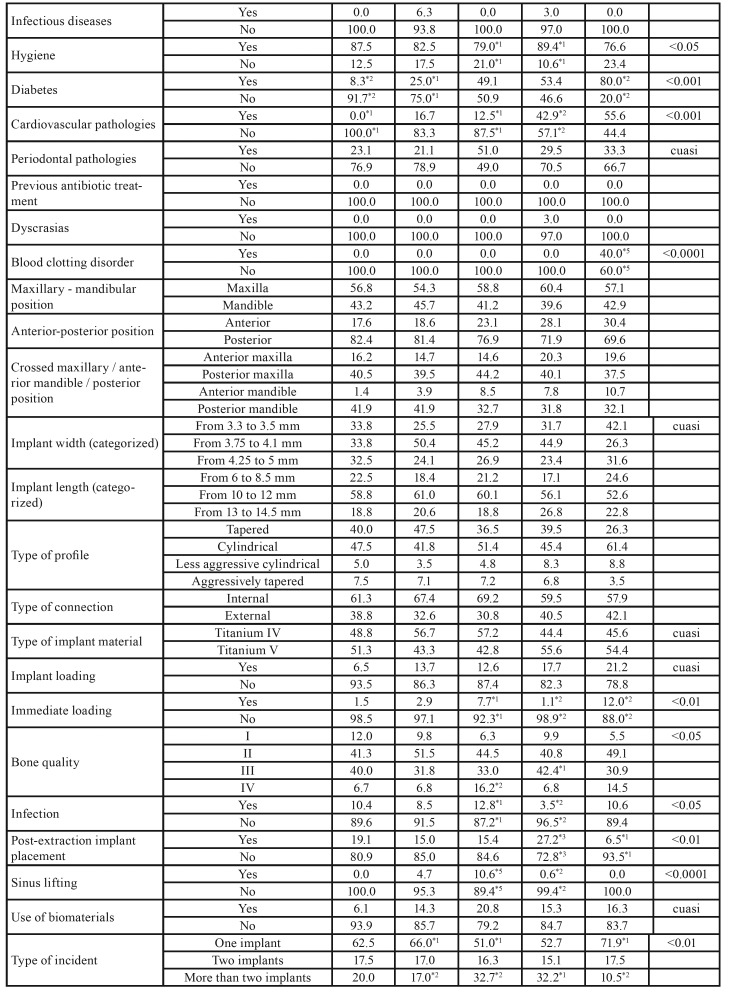
Crosstab analysis of age. Note: If the sum of the categories does not reach the value of 917, the differential corresponds to missing data.

**Table 4:**
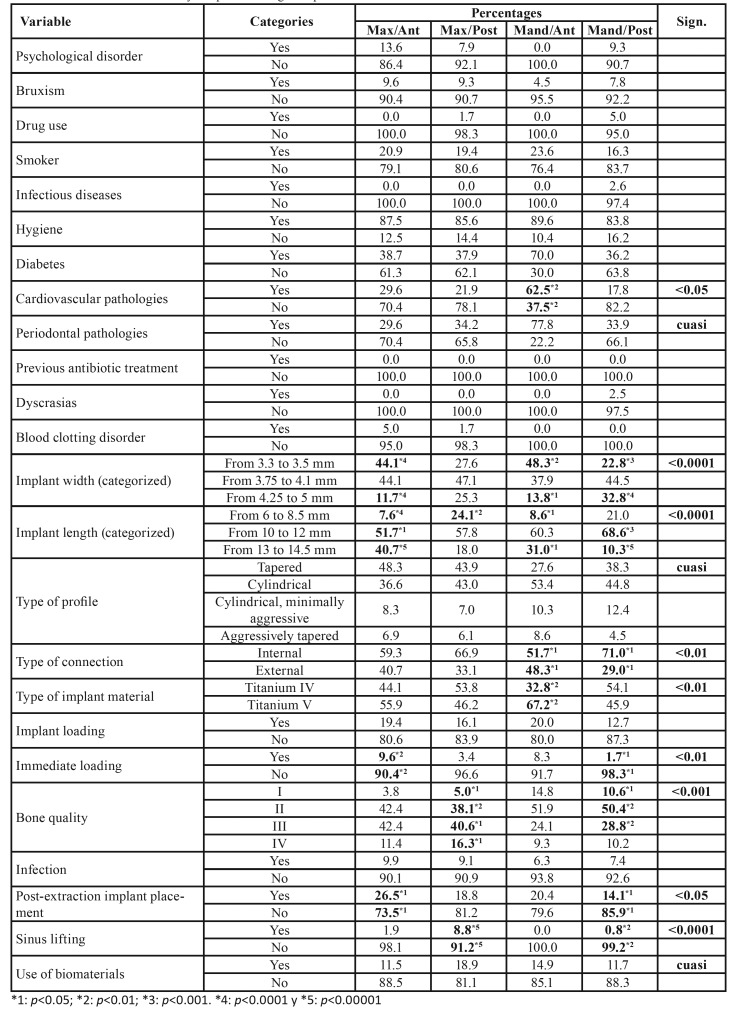
Characteristics of the study sample according to implant location.

Table 4 provides a detailed analysis of the variables collected in incident reports of implant failure according to implant position. Implant position was categorized into four groups: anterior maxilla, posterior maxilla, anterior mandible, and posterior mandible (Table 1). A higher incidence of implant failure was found in implants placed in the anterior maxilla with a length of 10 to 12 mm (*p* < 0.05) and 13 to 14.5 mm (*p* < 0.0001), in implants with immediate loading (*p* < 0.01), or in implants placed post-extraction (*p* < 0.05). Regarding implants placed in the posterior maxilla, a higher incidence of implant failure was observed when the implant length was 6 to 8.5 mm (*p* < 0.01), when placed in type II (*p* < 0.01) and type III (*p* < 0.05) bone quality, and when a sinus lifting had been previously performed (*p* < 0.00001). When analyzing the data regarding implants placed in the anterior mandible, a higher incidence of implant failure was observed in patients with cardiovascular pathologies (*p* < 0.01), in implants with a narrow diameter of 3.3 to 3.5 mm (*p* < 0.01), implants with a length of 13 to 14.5 mm (p < 0.05), implants with an internal connection (*p* < 0.05), and Grade V titanium implants (*p* < 0.01). The analysis of failed implants placed in the posterior mandible showed a higher incidence of implant failure in implants with a diameter of 4.25 to 5 mm (*p* < 0.0001), implants with a length of 10 to 12 mm (p < 0.001), internal connection implants (*p* < 0.05), and implants placed in type II bone quality (*p* < 0.01).

The implants with the highest rate of failure were tapered implants (3.5%; p < 0.0001), internal connection implants (2.5%; p < 0.0001), Grade IV titanium implants (3.6%; p < 0.0001), 3.3–3.5-mm narrow-diameter implants (2.6%; p < 0.001), and implants of 6 to 8.5 mm in length (2.9%; p < 0.0001) (Table 5).

**Table 5:**
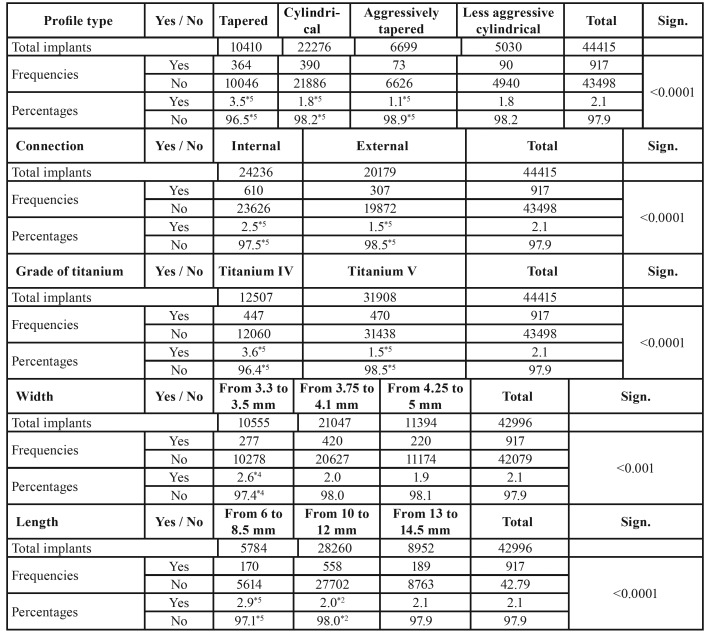
Significance of incident prevalence and type of complaint. Note: If the sum of the categories does not reach the value of 917, the differential corresponds to missing data.

## Discussion

Incident reports of implant failure can be considered as a powerful tool for descriptive analysis of the factors that most frequently appear in cases of implant treatment failure. While it is true that causal associations cannot be strictly established in these types of descriptive studies, as it is impossible to assess the entire population that has received all implants, they are nevertheless a tool to consider when evaluating the effectiveness and durability of a medical device.

Despite the usefulness, potential and, above all, the need for post-marketing control studies, this does not mean they are exempt of limitations. In this particular case, two such limitations can be cited as the most evident, and these must therefore be considered when interpreting the results. Firstly, in certain variables, the number of missing values may be important; clinicians collect data as part of their daily tasks rather than within the context of closed, reliable and controlled research protocols. This has the benefit of making pharmacovigilance studies applicable to a significant number of situations, which would otherwise be realistic if using only controlled prospective scientific studies. The second limitation is that the database belongs to a company with business interests. Although the present study has benefited from substantial and unlimited access to data records, it is important to recognize that they do not originate from a non-profit entity or from outside the company itself. In any case, the research team behind the present study was responsible for the handling and treatment of these data. In addition, this study has sought to extricate these data from any commercial interests, instead aiming to use them to increase scientific knowledge as much as possible.

The scientific evidence regarding age as a risk factor for implant failure is still up for debate. Several authors argue that there is no significant link between patient age and increased risk of implant failure (5-8). However, other published studies have found an increased risk of implant failure in patients over 60 years of age in comparison with patients older than 40 (1,9-11). In their 2017 study, Cars *et al*. observed a 7% increased risk of implant failure risk for every 10 years of age. In 2018, Lin *et al*. observed a higher risk of implant failure in patients older than 40. The present study observed the highest percentage of implant failure incident reports in the age range of 51–60 (30.1%) and 60–70 (29.7%).

When analyzing whether sex constitutes a risk factor for implant failure, there are discrepancies between the findings of different authors. Several researchers suggest that gender should not be considered a risk factor for implant failure (7,10,12,13). However, other authors have found a statistically significant higher risk of implant failure in males (8,14-19). Other studies have found a higher incidence of implant failure in women compared to men (1,20). In the present study, the highest percentage of incident reports of implant failure was observed in males (61.6%) rather than females (31.4%).

An analysis of the scientific literature reveals a correlation between males and other variables such as tobacco use or oral hygiene, as well as a higher risk of implant failure (19). In the present study, the incident reports of implant failure in patients who smoke showed a higher percentage of male smokers than females (*p* < 0.01), while the reports corresponding to non‑smoking patients showed a higher percentage of females than males (p < 0.01). With regard to oral hygiene, poor oral hygiene was more frequently observed in males than females (*p* <0.05).

Very few absolute medical contraindications have been suggested in the literature with regard to dental implant placement, but they include patients with recent myocardial infarction, heart valve surgery, risk of uncontrolled bleeding, or those who use intravenous bisphosphonates (21). Relative risk factors for early or late implant failure include tobacco use, diabetes mellitus, and compromised patient immunity (22). However, many of these studies used small sample sizes that make it difficult to extrapolate results to the general population (10), and there are also studies in which the presence of systemic pathologies was not correlated with a higher risk of implant failure (18). In the present study, a higher risk of implant failure was observed in patients over 70 with diabetes mellitus (p < 0.01), as well as in patients aged 61–70 with cardiovascular pathologies (p < 0.01).

The use of antibiotics prior to oral implant surgery results in a decrease in systemic bacteraemia after oral surgical procedures, along with a lower risk of implant failure (23). However, in their 2017 study, Hickin *et al*. (1) suggested that administration of antibiotics prior to implant placement surgery was not sufficient and should be complemented by postoperative administration in order to achieve maximum antibiotic coverage. In the present study, no previous antibiotics had been administered in any of the reported incidents of implant failure.

The occurrence of postsurgical infections is usually linked to poor oral hygiene due to an increase in bacterial biofilm and the subsequent infection of the area surrounding the implant, resulting in bone loss that leads to implant failure over the short or long term (24). In the present study, patients between 51 and 60 years of age comprised the group with the poorest oral hygiene (p < 0.05) and the highest rates of postoperative infection (p < 0.05). Good oral hygiene practices, both prior to dental implant placement surgery and with regular maintenance afterwards, are an important factor in the success of implant treatment (25).

Different factors influence the area in which a dental implant is placed: bone density, which varies according to the bone area, and occlusal forces sustained during chewing or occlusal trauma, which also differ according to the area in which an implant is placed (26). With regard to the position of the implant in the bone (maxilla/mandible, anterior/posterior), no differences were observed in the incident reports of implant failure in relation to sex or age. Scardina & Messina (27) found that hormonal imbalances in postmenopausal women could affect soft oral tissues and bone density. In the present study, no differences in bone quality were found between men and women in the reported incidents of implant failure. However, Type III bone quality was more frequently observed in incident reports of patients between 61 and 70 years (p < 0.05), while the highest percentage of Type IV bone quality was observed in the age group of 51–60 years (p < 0.01). In their 2017 study, Chrcanovic *et al*. (28) suggested that dental implants placed in Type III bone posed a higher risk of implant failure than those placed in Type II bone, and that the same was true of dental implants placed in Type IV bone in comparison with those placed in Type I, II, or III bone. However, the authors concluded that bone quality alone does not present a risk factor and should be considered as part of a whole.

Bone stability is directly related to bone density, which varies according to the bone area; the posterior maxilla is the area with the worst bone density, followed by the anterior maxilla, posterior mandible, and anterior mandible (19). In the present study, the highest incidence of implant failure was found in dental implants placed in the posterior mandible with type II bone quality (*p* < 0.01), results that match those found by Jem *et al*. 2017 (29). A higher incidence of implant failure was also observed in the posterior maxilla with type III (*p* < 0.05) and type II (*p* < 0.01) bone quality, with these being similar to the results found by other authors (7,30-32). However, other authors have found a higher incidence of implant failure in implants placed in the anterior mandible (19).

Implants placed in the posterior region are subjected to more undesirable forces due to chewing forces and lateral movements with cusp inclination; occlusal loads during occlusion are three times more intense in the posterior region than in the anterior region (33,34). When the influence of implant length or diameter on the stress concentration of the peri‑implant marginal bone is analyzed in the literature, it is observed that implant length is not considered a relevant factor (35). When analyzing implant length in the present study, the dental implants with the highest incidence of failure were implants of 6 to 8.5 mm in length placed in the posterior maxilla (*p* < 0.01), perhaps due to the poorer bone quality of the posterior maxilla associated with a higher crown-implant ratio (36), although other authors differ in this respect (37). In addition, a higher incidence of failure was observed in implants with a length of 10 to 12 mm placed in the posterior mandible (*p* < 0.001), and in implants with the same length and an internal connection (*p* < 0.0001). With regard to implants with a length of 13 to 14.5 mm, the highest incidence of failure was observed in those placed in the anterior maxilla (*p* < 0.0001).

Implant characteristics such as manufacturing material, dimensions and shape (diameter, length, and degree of taper), and the type of interface between abutment and implant are factors to bear in mind when predicting the likelihood of successful implant treatment (38).

The availability and volume of bone sometimes limit the placement of implants with regular diameter and length, necessitating the use of more advanced regenerative surgical techniques with a higher risk of complications (39,40). Nevertheless, short or narrow implants are increasingly used in cases of poor bone availability, especially in medically compromised patients in whom the use of regenerative surgical techniques may pose a higher risk of dental treatment failure and possible destabilization or exacerbation of pathological conditions (41).

However, other authors suggest that implant length is not a significant risk factor for implant failure (19). In the case of short implants, if bone loss is excessive, the biomechanics resulting from the crown-to-implant ratio may lead to overload and consequent implant treatment failure (42). Numerous studies consider short implants to be a risk factor for implant failure (6,8,11,17,43-47). The present study found a higher incidence of implant failure in implants between 6–8.5 mm in length (p < 0.0001).

Regarding implant diameter, several authors suggest that narrow-diameter implants present a higher risk of prosthetic complications (48) or an increased risk of implant treatment failure (6). However, other authors have found no significant differences in the success of implant treatment when using narrow implants rather than regular-diameter implants (34,46,49-51). Recently, the literature has made reference to new surface treatments, alloys and manufacturing materials used for dental implants in order to improve their resistance and load capacity (52). Narrow-diameter implants manufactured with these new innovations have been shown to produce the same optimal results as regular implants (31,43). Other studies have found that implants with larger diameter (5 mm) have a higher risk implant failure than implants with a narrow or regular diameter (30,42). This may be because the area in which they were placed has a lower bone density, due to the characteristics of the implant design, or the implant bed (19). In the present study, a higher incidence of implant failure was observed in implants with a diameter of 3.3–3.5 mm (p < 0.001).

Another factor to consider when evaluating the likelihood of a successful implant treatment is the shape of dental implants, as this plays a vital role in how mechanical stress is distributed to the surrounding bone and the implant itself (34). In 1998, Holmgren *et al*. (53) reported that tapered implants caused greater stress on the bone crest that cylindrical implants of the same diameter; these results were later replicated by Tabrizi *et al*. in 2017 (34). In the present study, tapered implants had the highest rate of implant failure (p < 0.0001).

The implants placed in the present study were classified into two groups: implants made of pure Grade IV titanium (0.4% oxygen content) and those made of Grade V titanium, which is a titanium alloy (90%) with aluminum (6%) and vanadium (4%) that provides better resistance to stress fracture than pure Grade IV titanium (54). Pure Grade IV titanium implants had the highest percentage of failures in the incident reports examined (p < 0.0001). Few studies exist in the literature have evaluated the long-term success of dental implants with different titanium grades. In 2015, Hirata *et al*. (55) found that Grade V dental implants had better resistance, stability, and load distribution than dental implants manufactured with Grade II titanium, although the attachment fracture modulus was similar for both implants.

The effects of different connection types and the titanium grades that make up dental implants have been only briefly addressed in the scientific literature. In 2016, Park *et al*. (56) found no significant differences between implants with internal or external connections and Grade IV titanium from the same manufacturer, nor did they observe any differences between implants with similar internal connections and Grade IV titanium from two different manufacturers. However, there were significant differences between implants with Morse connections and internal connections made of the same Grade II titanium. They concluded that the use of a deeper internal connection and Grade IV titanium in the implant system is favorable to mechanical static overload, and when Grade II titanium dental implants are used, a larger diameter should be selected for the connection in order to provide sufficient strength to withstand the overload. The present study found that tapered implants had the highest rate of implant failure (p < 0.0001).

With regard to post-extraction implant placement, the literature shows that there are no significant differences observed between immediate and deferred implant placement (1,57). The present study found a higher percentage of incident reports of men who had received post-extraction implants than women (p < 0.05).

For some researchers, the use of bone regeneration surgical techniques may pose a higher risk of implant failure regardless of the graft material used during the procedure (17,19). Other authors have found that there is no such (11,58,59). In the present study, a higher percentage of implant failure incidents was observed in female patients with implant placement simultaneously after sinus lifting, in contrast with male patients (p < 0.05).

Another variable analyzed was the number of failed implants reported within the same incident report. Several authors state that the more implants placed during surgery, the higher the risk of implant failure (29,60). The reason for this may be due to the surgical area covered when placing more than one implant, which could affect blood supply to the area, increase surgical time, and result in greater likelihood of wound contamination. However, other authors have failed to find a correlation between number of implants placed and higher risks of implant treatment failure (19). In the present study, a higher percentage of failure of one placed implant was observed in females (p < 0.01) and in patients older than 70 (p < 0.05). On the other hand, a higher rate of failure of two implants was observed in males (p < 0.01). In the case of incident reports in which more than two implants had failed, a higher percentage was observed in patients aged between 51 and 60 (p < 0.01) and 61 and 70 years (p < 0.05).

As final conclusion, we can say that the analysis of the implants included in our study revealed a high rate of complications in short implants, tapered, internal connection and narrow. The knowledge of these data can help dentists to apply safer protocols when using these types of implants.
